# Vestibular Schwannoma and Tinnitus: A Systematic Review of Microsurgery Compared to Gamma Knife Radiosurgery

**DOI:** 10.3390/jcm13113065

**Published:** 2024-05-23

**Authors:** Ava M. King, Jaimee N. Cooper, Karina Oganezova, Jeenu Mittal, Keelin McKenna, Dimitri A. Godur, Max Zalta, Ali A. Danesh, Rahul Mittal, Adrien A. Eshraghi

**Affiliations:** 1 Department of Otolaryngology, Hearing Research and Cochlear Implant Laboratory, University of Miami Miller School of Medicine, Miami, FL 33136, USA; amk265@med.miami.edu (A.M.K.); jcooper12@student.touro.edu (J.N.C.); j.mittal@med.miami.edu (J.M.); kmcke018@med.fiu.edu (K.M.); dgodur@med.miami.edu (D.A.G.); 77mrzalta@gmail.com (M.Z.); danesh@health.fau.edu (A.A.D.); r.mittal11@med.miami.edu (R.M.); 2 School of Medicine, New York Medical College, Valhalla, NY 10595, USA; 3 Department of Communication Sciences and Disorders, Florida Atlantic University, Boca Raton, FL 33431, USA; 4 Department of Integrated Medical Sciences, Schmidt College of Medicine, Florida Atlantic University, Boca Raton, FL 33431, USA; 5 Department of Neurological Surgery, University of Miami Miller School of Medicine, Miami, FL 33136, USA; 6 Department of Biomedical Engineering, University of Miami, Coral Gables, FL 33143, USA

**Keywords:** vestibular schwannoma, tinnitus, gamma knife, microsurgery, surgical resection

## Abstract

**Background**: Vestibular schwannoma (VS) is a benign tumor of the eighth cranial nerve formed from neoplastic Schwann cells. Although VS can cause a variety of symptoms, tinnitus is one of the most distressing symptoms for patients and can greatly impact quality of life. The objective of this systematic review is to comprehensively examine and compare the outcomes related to tinnitus in patients undergoing treatment for VS. Specifically, it evaluates patient experiences with tinnitus following the removal of VS using the various surgical approaches of traditional surgical resection and gamma knife radiosurgery (GKS). By delving into various aspects such as the severity of tinnitus post-treatment, the duration of symptom relief, patient quality of life, new onset of tinnitus after VS treatment, and any potential complications or side effects, this review aims to provide a detailed analysis of VS treatment on tinnitus outcomes. **Methods**: Following PRISMA guidelines, articles were included from PubMed, Science Direct, Scopus, and EMBASE. Quality assessment and risk of bias analysis were performed using a ROBINS-I tool. **Results**: Although VS-associated tinnitus is variable in its intensity and persistence post-resection, there was a trend towards a decreased tinnitus burden in patients. Irrespective of the surgical approach or the treatment with GKS, there were cases of persistent or worsened tinnitus within the studied cohorts. **Conclusion**: The findings of this systematic review highlight the complex relationship between VS resection and tinnitus outcomes. These findings underscore the need for individualized patient counseling and tailored treatment approaches in managing VS-associated tinnitus. The findings of this systematic review may help in guiding clinicians towards making more informed and personalized healthcare decisions. Further studies must be completed to fill gaps in the current literature.

## 1. Introduction

**Figure 1:**
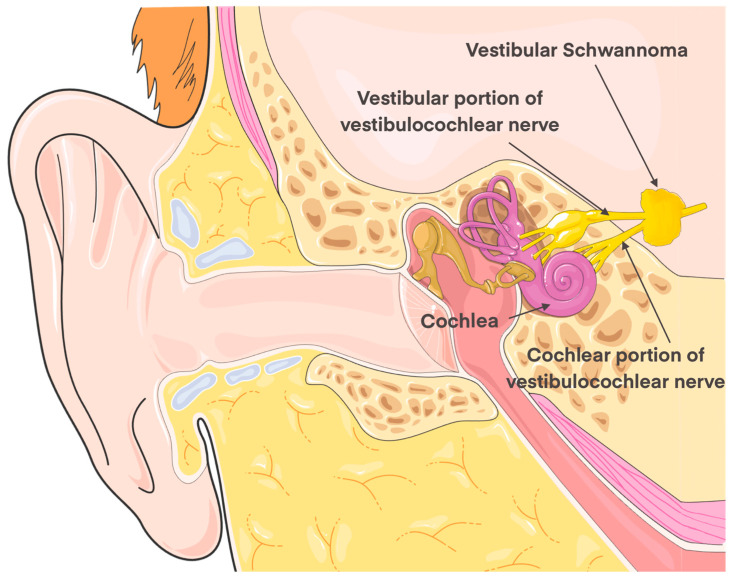
A schematic representation of vestibular schwannoma (VS). The two portions of the eighth cranial nerve (vestibulocochlear nerve) are highlighted along with a representation of VS. The figure was generated using images from Servier Medical Art, provided by Servier, licensed under a Creative Commons Attribution 4.0 unported license.

Vestibular schwannomas (VSs), also known as acoustic neuromas, are benign tumors of Schwann cells that originate from CN VIII (vestibulocochlear nerve), commonly the vestibular branch of CN VIII [1] (Figure 1). In 2020, VS had a prevalence between 3 and 5.2 per 100,000 person years which has increased over the past 20 years [2,3]. Despite being benign, these tumors may affect patients over time as they grow, by causing tinnitus, unilateral sensorineural hearing loss, vestibular symptoms, or cranial nerve deficits, such as CN VII (facial nerve) palsy [4,5]. Up to 95% of VSs are unilateral, sporadic, and occur in the fifth to seventh decades of life, although this may also be seen in those with neurofibromatosis type 2 (NF2), where VSs often present bilaterally in these patients [6,7]. The symptoms of VS can be distressing and may be correlated with other symptoms such as depression and anxiety [8,9,10]. VSs are often classified using the Koos grading system, which uses numbers I through IV [11]. An increasing tumor score is helpful for surgical planning as well as assessing postoperative complications. While watchful observation may be suitable for the management of some cases of VS, prompt treatment is the standard of care in those presenting with bothersome symptoms or growing tumor size to alleviate any symptoms and to prevent further damage to nearby structures as the tumor grows [11].

Treatment modalities for VS include surgical resection and radiosurgery (such as gamma knife radio surgery) [1,12]. GKS for VS has been popularized in the last decade, although empirical data on the best approach are still lacking [11,12]. Surgical intervention for VS is a safe procedure with low mortality (0.38%) and complication rates (5.3%) [13]. GKS also has shown itself to be safe and effective with sources citing tumor control in up to 97.1% of patients with very low complication rates [14]. For surgical resection, the approach can influence the possible complications; there are three approaches for VS resection, translabyrinthine (TL), middle fossa (MF), and retrosigmoid (RS) [1,15]. The TL approach results in complete hearing loss due to obliteration of inner ear structures, but generally allows for total excision of the mass with a decreased chance of facial nerve injury. Although the eighth cranial nerve is usually completely resected, there is no guarantee that tinnitus will be entirely eliminated when the TL approach is used. The MF approach yields the best hearing preservation, but requires temporal lobe retraction, may only be used on smaller tumors, and leaves the possibility of tumor regrowth in unfavorable locations. The RS approach has the potential for hearing preservation and can be used on any-sized tumors [16]. Chang et al. retrospectively studied the outcomes of GKS on large VS tumors (>8 cc) [17]. While 66.7% experienced positive outcomes, it was observed that some patients developed new postoperative issues, including trigeminal nerve dysfunction, hydrocephalus, imbalance and unsteady gait as well as a decrease in sensory perception [17]. The complications of these management options have been well studied; however, the literature specifically related to tinnitus is more limited.

Tinnitus is often described as a persistent ringing or buzzing sound. Subjective tinnitus, the focus of this study, is a phantom sound which occurs in the absence of any external sensory stimulus (as tinnitus can be evoked by orofacial, gaze, and somatosensory stimulations) that may be transient or constant [18]. Objective tinnitus is a sound that can be heard by others. Patients often find it distracting and disruptive, especially since treatment of tinnitus is often ineffective [19,20]. Tinnitus, with regard to VS, may be present in 63–75% of patients undergoing surgical resection [21,22,23,24]. The pathophysiology of tinnitus in VS is not fully elucidated with many theories on its origin. Tinnitus in VS may be due to compression of the vestibulocochlear nerve, ischemia within the cochlea, or other dysfunctions of the neural system between the inner ear (cochlea) and the auditory centers of the brain [25]. Later in the course of having tinnitus it may centralize, meaning the pathology is occurring in the brain; when this happens, we have limited ability to treat the tinnitus itself and treatment shifts to cognitive therapies and audiological management such as sound therapy to decrease the distress caused by the tinnitus. While VSs are a potential cause of tinnitus, there are many other possible etiologies, such as chronic exposure to loud noises, Meniere’s disease, ototoxic medications, aging, metabolic disorders, and idiopathic etiologies [26].

The primary objective of this systematic review article is to evaluate the impact of VS resection or GKS on tinnitus outcomes. By conducting a comprehensive review of traditional surgical resection methods and GKS, this review seeks to highlight the differences in efficacy and patient experiences relating to tinnitus following VS removal. This systematic review aims to contribute significantly to the body of knowledge on VS management, specifically in relation to tinnitus outcomes, and to facilitate in the decision-making process for both healthcare professionals and patients considering surgery for VS.

## 2. Materials and Methods

### 2.1. Search Strategy

A protocol for this systematic review was developed a priori and registered in the PROSPERO database (registration number: CRD42023439127). Searches were performed in PubMed, Science Direct, Scopus, and EMBASE databases. Searches were conducted using the following terms: “vestibular schwannoma treatment AND tinnitus”; “vestibular schwannoma AND hearing loss”; “vestibular schwannoma and hearing loss and tinnitus”; “vestibular schwannoma AND surgical resection”; “vestibular schwannoma AND surgical resection AND tinnitus”; “vestibular schwannoma AND gamma knife radiosurgery”; “vestibular schwannoma AND gamma knife radiosurgery AND tinnitus”.

### 2.2. Study Selection

All relevant search results, including titles, abstracts, and full texts, were reviewed independently by two researchers. Disagreements over inclusion and exclusion were resolved by a discussion with other researchers involved in the study. Exclusion criteria included meta-analyses, abstract only, review articles, editorials, and those with outcomes not related to this study topic. Studies not in the English language and animal studies were also excluded. In an effort to focus on recent literature, articles from before the year 2016 were excluded. Articles were reviewed first by abstract and then by full text if deemed relevant in relation to VS and tinnitus.

### 2.3. Data Extraction

Two investigators (A.K., J.C.) independently reviewed the included articles. The information gathered includes: study type, population, comparison/study, surgical approach, pre- and post-operative tinnitus, and outcomes/conclusions. After initial data extraction, each investigator gathered additional information on other VS symptoms or surgical outcomes. Analysis of tinnitus outcomes in the microsurgery and GKS cohorts was also performed.

### 2.4. Quality Assessment

The ROBINS-I (Risk of Bias In Non-randomized Studies-of Interventions) tool was used to assess the risk of bias [27,28]. The ROBINS-I assesses seven domains whereby bias may arise (confounding, selection of participants, classification of interventions, deviations from intended interventions, missing data, measurement of outcomes, and selection of the reported result). The guidelines for the ROBINS-I tool were utilized in formulating domain specific and overall judgements on bias [28]. This was completed by one investigator and evaluated by a second investigator. Any disagreements were discussed with other research investigators. The appropriate checklist was utilized based on the type of study. This assessment was completed by two reviewers (J.C., A.K.) independently, with discrepancies resolved by discussion and consensus or discussion with the senior author [6,19,29,30,31,32,33,34,35].

## 3. Results

### 3.1. Study Selection

**Figure 2:**
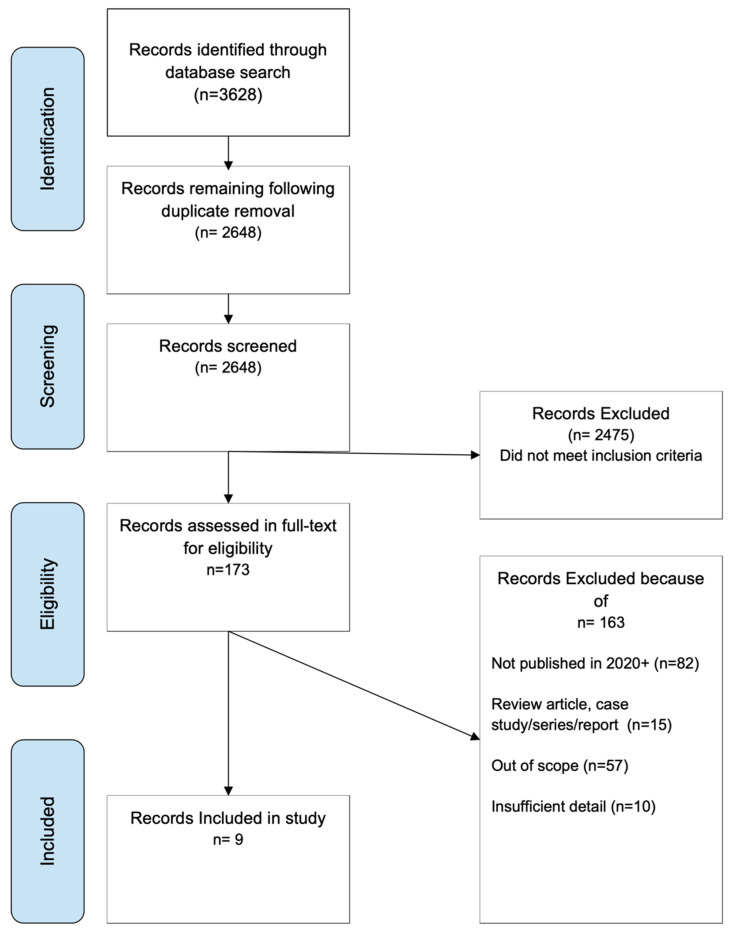
PRISMA diagram detailing the inclusion and exclusion process of search results.

Using the search terms on each database, 3629 papers were extracted. After duplicates were removed, 2648 papers remained. This number was narrowed down to 173 using the exclusion criteria. After full-text reviews, 10 papers were selected for the review after being deemed relevant for this study (Figure 2). A summary of all included studies is shown in Table 1.

### 3.2. Quality Assessment of Included Studies

**Figure 3:**
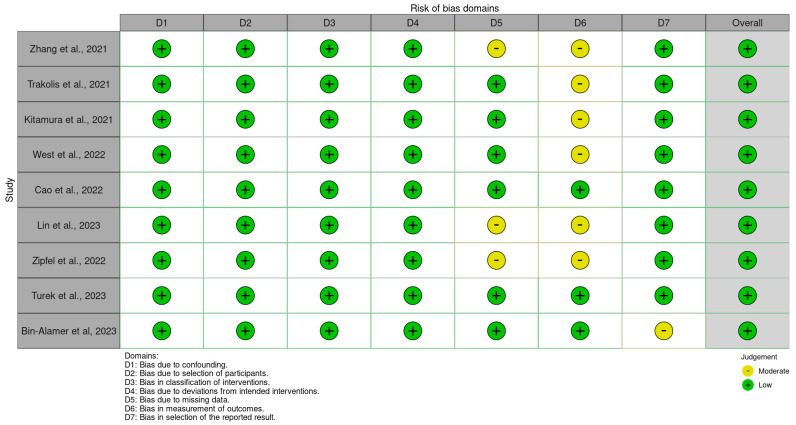
Risk of bias analysis utilizing the ROBINS-I tool [6,19,29,30,31,32,33,35,36].

The risk of bias (RoB) analysis was conducted using the ROBINS-I tool. Figure 3 shows the risk of bias assessment performed on the included studies.

### 3.3. Surgical Resection

Zhang et al. conducted a study on 237 patients who underwent VS surgery and had pre-operative tinnitus, and 90 patients who did not have tinnitus [29]. Translabyrinthine (TL) and retro sigmoid (RS) approaches were used in the included patients. Initially, 72.5% of patients had tinnitus preoperatively leaving 27.5% without it. Of those that began with tinnitus, 44.7% were improved after surgery, 35.9% were unchanged, and 19.4% reported worsened tinnitus. Of those with no tinnitus preoperatively, 22.2% developed tinnitus post-operatively. While demographics, age, gender, and tumor size were not correlated with tinnitus outcomes, the TL approach had a statistically significant difference in tinnitus improvement when compared to RS, 48.4% versus 37.2% as well as tinnitus worsening 15.1% vs. 28.2,% respectively. The same was seen in those with no preoperative tinnitus regarding the surgical approach; new onset tinnitus was 15.9% vs. 37.0% with TL and RS approaches, respectively. This study, being retrospective in nature, inherently contains biases. This is particularly due to the fact that patients were inquired about their postoperative tinnitus anywhere from 1 to 9 years following the surgery, in cases where it was not previously recorded.

Trakolis et al. studied 46 patients with spontaneous VSs who underwent surgical treatment and had sustained or ceased tinnitus postoperatively [30]. Preoperatively, 57% of the cohort had tinnitus with 22% of those with preoperative tinnitus having persistent tinnitus postoperatively and 62% having cessation of the tinnitus postoperatively. Overall, 26% of the total cohort had tinnitus postoperatively. Sustained tinnitus was defined as tinnitus persisting for at least 3 months postoperatively. They were specifically interested in changes in the brain in these cohorts as measured by surface-based morphometry. Those with persistent tinnitus ≥ 3 months, had a grey matter increase in the contralateral fusiform gyrus, contralateral middle temporal gyrus, contralateral medial frontal gyrus, ipsilateral superior colliculus, and an ipsilateral and contralateral caudate nucleus. They concluded that tinnitus that persisted after surgery (≥3 months) was likely due to centralization, which is reflected in the morphometry results.

Kitamura et al. analyzed 71 patients who underwent VS resection. Of the 71 participants, 63.3% did not require treatment for tinnitus, and 36.6% of patients had at least one episode of tinnitus distress, defined as a tinnitus handicap index (THI) > 18 [31]. Preoperatively, the mean THI score was 15.8 with a range of 0–62. Postoperatively, the mean THI score was 10.1, with a range of 0–50, at a mean of 34.7 months post operation. Tinnitus results were also analyzed by the surgical approach: retrolabyrinthine approach (RL), middle fossa approach (MF), or translabyrinthine approach (TL). RL had a mean THI 7.9, MF had a mean THI of 12.9, and TL had a mean of 10.7; however, these differences were not statistically significant, but the trend was for MF having worse tinnitus outcomes. For treatment of the tinnitus, educational materials were first given; if no improvement was seen, further intervention was offered. Of the 17 patients who only received educational materials, the median worst THI score was 33.9 and the latest score was 22.8, which was found to be a statistically significant result. For the 9 patients that received further intervention for tinnitus, two received SSRIs, with hearing aids (HA) alone given to the remaining 7. For the SSRI group, they both had significant anxiety and depression with original mean THI scores of 42 and an improved median score of 5. For those that received HA, prior to HA median scores were 28.9, and 9.7 postoperatively. The results of those who received SSRIs should be further studied as a patient population of two is not sufficient to conclude their utility [31].

West et al. analyzed the tinnitus burden, via THI, of 22 patients with VS who subsequently received an ipsilateral cochlear implant (CI) [32]. Both pre- and postoperative THI were available for 17 patients; there were only postoperative scores in two patients, and one patient had a qualitative report of severe tinnitus preoperatively. Seventeen cases underwent cochlear implantation, with 77% of all patients having a reduction in the tinnitus burden, 18% with no change, and 6% with worsened tinnitus. The preimplantation average THI score was 18 and postoperatively the average was 10. Their conclusions showed that the majority of individuals who received a CI after VS resection had a decrease in their THI. The area of the THI that saw the most improvement was the functional domain, followed by the catastrophic domain.

Cao et al. analyzed 401 patients with VS who underwent TL or RS resection. A total of 67.4% of the cohort had preoperative tinnitus. They found no significant association between the surgical approach and tinnitus outcomes; however, female gender and tumor size >15 mm were found to be independent factors associated with good tinnitus outcomes [33]. This is in line with prior studies which have indicated sex differences in patients with VS [36].

Lin et al., specifically, studied tinnitus outcomes in patients with unilateral VSs [19]. A total of 32 VS patients were compared to controls using MR imaging in order to conduct functional hierarchy analysis. In the included patients, the VSs were treated via resection using the retrosigmoid approach. For the evaluation of tinnitus, researchers utilized the THI and the visual analog scale (VAS). Eighteen of the 32 included participants had tinnitus. There was no statistical significance of the presence of tinnitus in regard to gender, age, side, and preoperative hearing. The researchers found that only one participant showed complete resolution of tinnitus following surgical resection, with five showing improvement in their tinnitus. Some patients did note worsening tinnitus, and some patients with no preoperative tinnitus subsequently acquired tinnitus in the postoperative period. There was no factor, such as demographics or tumor characteristics, that was found to be statistically significant regarding changes in tinnitus postoperatively. The researchers also studied global gradients via rs-fMRI and their possible relation to tinnitus in these patients. Global gradients are an organizational system for the study of the brain; they refer to the differences in function and organization of the brain under various conditions [37,38]. Multiple gradient distances were statistically significant in their relation to tinnitus. For example, the RH_DorsAtten_post_24 gradient is related to the vestibular system, and interference could contribute to tinnitus preservation in patients with VSs. Specifically, the researchers discovered that in the postcentral gyrus region of the brain, changes to the functional gradient may have a connection to tinnitus in those suffering from VS. A limitation of this study is the small sample size. In addition, it would be helpful to have more follow-up with participants.

Zipfel et al. studied the outcomes in 28 VS patients at less than 21 years of age following retrosigmoid surgical resection, aged 16 ± 3.3 years [34]. Looking at the tinnitus outcomes of this study, we see that 39.3% of study participants had tinnitus preoperatively. This number decreased to 10.7% postoperatively. The researchers deemed that the resection of sporadic VS in the pediatric population is an appropriate treatment option. As with many studies, one limitation is the small sample size of 28 participants. More studies should be conducted in the pediatric realm on this topic to garner more solid evidence on outcomes in this population.

### 3.4. Gamma Knife Radiosurgery

Turek et al. conducted a prospective cohort study of 94 patients with intracanalicular VSs to study patient outcomes status post radiosurgery treatment (gamma knife stereotactic radiation (GKS) [35]. After undergoing treatment, each patient was scheduled for a follow-up MRI 12 months later. However, a group of 16 patients underwent follow-up MRI sooner due to intensified symptoms of headache and tinnitus. Interestingly, 10/16 of these patients were labeled as having pseudoprogression, which is classified as an increased VS tumor volume on early postoperative MRI which subsequently decreases in size on subsequent MRIs. The researchers found that there was an overall decrease in tinnitus burden post-GKS. A total of 62 patients experienced tinnitus prior to GKS, and 57 continued to experience tinnitus at the end of the study. The decrease in the number of patients experiencing tinnitus status post GKS did not reach statistical significance in the study. This study focused on tumor characteristics, hearing, and facial nerve preservation, with a brief discussion of tinnitus outcomes.

In a study by Bin-Alamer et al., the authors studied patient outcomes after undergoing stereotactic radiosurgery for the removal of VSs in patients with NF2 [6]. VSs are commonly seen in patients with NF2. The specific aim of this paper was to study outcomes including control of the tumor(s), hearing preservation, freedom from additional treatment, and tumor development or transformation following stereotactic radiosurgery. Tinnitus was present in 121 patients included in the study. Following treatment, 103 of these patients saw no improvement in their tinnitus. Of the 18 patients with changes in their tinnitus, only 6 showed improvements, with the remaining 12 patients having worsening of their tinnitus. With reference to tinnitus, a limitation of this study is that it does not further describe tinnitus outcomes. See Table 1 for an overview of each article.

## 4. Discussion

### 4.1. Benefits and Disadvantages of Gamma Knife VS Surgical Resection

The first surgical resection of a VS was performed in the early 1900s. With advances in surgical techniques, the approaches used have evolved with the creation of more precise imaging and new tools [39]. Open surgical techniques were the standard until the 1990s when the use of GKS was introduced as a treatment modality for VS and gained popularity among certain patient populations [40]. Numerous studies have evaluated GKS VS surgical resection as techniques for management of VS [41,42]. Most of these papers focus on hearing preservation or facial nerve function outcomes, rather than tinnitus [43,44]. For example, Han et al. compared the outcomes of microsurgery (MS) VS GKS over 26 years. They found that GKS is better for older patients with AAO-HNS Class A hearing loss preoperatively and in worse physical shape. There are previous studies investigating the patient populations who most benefit from GKS, which includes these older adults and those with smaller tumor sizes or for those who have undergone prior resection with regrowth or incomplete removal [41,45]. MS should be considered in younger patients who are in good shape physically, with acceptable preoperative hearing due to the higher chance of hearing preservation in the long term [46]. In another study, Sun et al. retrospectively studied patients with VS who underwent GKS and found that hearing preservation is lower in those with larger tumors [47]. Another study by Park et al. did look at tinnitus in translabyrinthine microsurgery (TLM) VS GKS [48]. The researchers discovered that after TLM, tinnitus improvement rates were higher than after GKS (*p* = 0.016) evaluated through THI scores. Within the GKS group, there was worsening of tinnitus (*p* < 0.001), which was also based on THI scores and was seen in VAS. Looking at mean THI scores, there was a significant reduction postoperatively within the TLM group (*p* = 0.006). On the other hand, the GKS group showed increased THI mean scores postoperatively (*p* < 0.001). The researchers found in this study that TLM with vestibulocochlear neurectomy could help with tinnitus levels [48]. On the other hand, Baguley et al. discovered that tinnitus is neither made worse nor made better by the translabyrinthine excisional approach [49]. Boari et al. had interesting findings regarding outcomes after GKS for VS patients. Significantly, in patients with ≥2 h of using a mobile phone every day, there was more of a chance of tinnitus following GKS (*p* = 0.036). In addition, within the internal acoustic canal, the length of the VS within the IAC predicted the occurrence (*p* = 0.047) and persistence of tinnitus following GKS (*p* = 0.029). Looking into hearing, they discovered that patients at or older than 55 years of age had a higher chance of losing serviceable hearing after GKS (*p* = 0.014) [14]. The scientific evidence on MS versus GKS approaches is heterogeneous in terms of patient populations and tumor characteristics that are optimal for each technique, and direct comparison of the surgical approaches is required. Patient age and tumor size are the main clinical factors which may influence the decision between surgical resection and GKS. The percentage of patients with tinnitus before and after microsurgery or GKS was similar in this study and does not appear to influence the clinical decision-making between these two modalities. West et al. were the only authors to include cochlear implantation in their study. CIs appear to reduce the tinnitus burden in that cohort; however, further investigation into the role of CIs in VS treatment is needed to reach a conclusion. Patient preference plays a huge role in deciding the course of treatment and should be taken into account along with their other health factors and tumor characteristics [50,51,52]. Both GKS and MS are widely used to treat VSs. With regard to tinnitus outcomes, there was no discernible difference between the two approaches.

### 4.2. Spontaneous Schwannoma VS NF2

VSs generally present spontaneously, with a prevalence of more than 1 per 500 patients, and usually unilaterally [2,53]. NF2, a genetic disease associated with bilateral VSs as well as other tumors at various locations (schwannomas, ependymomas, meningiomas), has a prevalence of around 1 in 60,000 people [6,54]. Patients with NF2 generally receive screening for VS at diagnosis, leading to earlier diagnosis, smaller tumor sizes, and a more aggressive course of treatment [55]. Tinnitus is a common presenting symptom in both types of VS patients. Up to 75% of patients with the sporadic form and 80% of patients with NF2-associated VS have tinnitus [15,55,56]. In a retrospective study, Naros et al. found that most patients (61.8%) in their cohort of patients with sporadic and unilateral VSs presented with tinnitus prior to surgical management [22]. Interestingly, they found that those with preoperative hearing had a higher risk of developing postoperative tinnitus, compared to those with preoperative deafness. Alternatively, Tosi et al. studied patients with NF2 undergoing radiosurgery for the removal of VS(s) [57]. Just as is seen in NF2 associated VS, patients with NF2 may also present with tinnitus as an initial symptom, although it may be less common due to increased surveillance and earlier detection [57]. Sporadic and NF2-related VSs are frequently studied separately due to differences in tumor behavior, including their location (in the IAC and CPA), their appearance (lobular in NF2), and adherence to local structures [55,58]. In NF2-associated VSs, the tumors generally have a higher proliferation index and varied characteristics depending on the NF2 mutation [59].

### 4.3. Age

Another potentially significant factor to consider in studies of tinnitus is the patients’ age at diagnosis and treatment. There is no consensus on the effect or correlation of age on the tinnitus burden; there are studies which find that age is not correlated with the tinnitus burden pre- or postoperatively [60,61], while other studies h have found that tinnitus intensity was positively correlated to age in a group of patients with VS [62,63].

## 5. Limitations

This systematic review includes retrospective studies, which come with their inherent limitations. Tinnitus is a difficult entity to evaluate as many of the tests rely on subjective recounts of the tinnitus burden. Additionally, some of the included studies also relied on retrospective evaluation of the tinnitus burden which introduces recall bias. An additional limitation of this study was the smaller number of papers which fulfilled all inclusion and exclusion criterion specifically regarding tinnitus outcomes in patients undergoing GKS, leading to insufficient data for comprehensive analysis of outcomes. In studies involving surgical outcomes, studies reporting complications or negative outcomes tend to be underrepresented [64]. The paucity of directly relevant studies underscores the need for more focused research in this area to better understand the impact of VS resection via microsurgery and GKS on tinnitus outcomes.

In addition, the evidence on surgical approaches versus gamma knife surgery (GKS) is highly heterogeneous, particularly concerning patient populations and tumor sizes, complicating any direct comparisons between the included studies. The diversity in patient demographics, such as age, underlying health conditions, and tumor characteristics (including size, location, and type), creates significant variability in the outcomes observed. This variability makes it challenging to draw definitive conclusions about the tinnitus outcomes in surgical approaches and GKS. Therefore, comprehensive studies that systematically compare these modalities while rigorously controlling for confounding variables are essential. The information derived from these studies will help us to understand the effects of the treatment modalities themselves, providing clearer insights into their comparative benefits and risks, ultimately guiding better clinical decision-making.

## 6. Conclusions and Future Directions

Our study identified significant gaps in the literature concerning the outcomes of tinnitus in patients with VS, emphasizing the need for a more thorough examination of the issue. We found a wide variation in how studies reported tinnitus outcomes, making it difficult to conduct a comprehensive statistical analysis. For example, some studies measured tinnitus using pre- and postoperative THI scores; others categorized improvements or worsening, and some only noted the presence of tinnitus after surgery without detailing any qualitative changes.

Our findings suggest that the presence of tinnitus alone should not dictate the choice of treatment for VS. It is crucial to understand these varied tinnitus outcomes to manage patient expectations effectively and to improve treatment approaches. This research is a preliminary step towards refining clinical decision-making for treating VS, whether through surgical resection or GKS, by compiling and analyzing surgery and GKS outcomes related to tinnitus.

Continued research in this area is vital for enhancing our knowledge and improving the treatment results for patients with VS and tinnitus. Future studies should focus on large, well-defined patient groups and use uniform reporting methods to allow for meaningful statistical analyses. Additionally, studying patients who opt for watchful waiting could provide insights into the natural progression of tinnitus and help refine treatment strategies that include microsurgery and/or GKS. The potential of future research extends beyond scientific discovery; it also promises to improve the quality of life for those with VS-related tinnitus.

## Figures and Tables

**Table 1:** A summary of included studies.

Reference	Study	Specialty	Population	Comparison/Study	Outcomes
Zhang et al. [29], 2021	Retrospective Cohort	Otolaryngology	237 patients who underwent surgical repair of a vestibular schwannoma with preop tinnitus and 90 VS patients without tinnitus.	Postoperative tinnitus and hearing outcomes of patients who got VS surgery.	In those with preoperative tinnitus, the tinnitus improved in 44.7% of patients, worsened in 19.4%, and remained the same in 35.9%. In those without preoperative tinnitus.
Trakolis et al. [30], 2021	Cross-Sectional	Neurosurgery	46 unilateral spontaneous VS patients with sustained or ceased tinnitus after surgery.	Cortical structural changes in those with sustained or ceased tinnitus after surgery with high resolution MRI.	57% of the patients had preoperative tinnitus, and 62% of these cases had cessation of tinnitus postoperatively. Conversely, 22% of patients with preoperative tinnitus had it continue for more than three months after surgery.
Kitamura et al. [31], 2021	Prospective Cohort	Otolaryngology	71 patients who underwent surgical resection of a VS.	Tinnitus severities (via the THI) relationship to surgical approach and hearing outcome and THI changes over time.	63% of participants did not require treatment for tinnitus, and 36.6% patients had at least one episode of tinnitus distress (THI > 18). There was no significant correlation between surgical approach and residual hearing on tinnitus severity. Preoperatively the mean THI score was 15.8 with a range of 0–62. Postoperatively, the mean THI score was 10.1 (range 0–50) at a mean of 34.7 months (range 8–71 months). When stratified by surgical approach, RS had a mean THI 7.9, MF had a mean THI of 12.9, TL had a mean of 10.7, however these differences were not statistically significant.
West et al. [32], 2022	Retrospective Cohort	Otolaryngology	22 consecutive patients with VS who received an ipsilateral CI.	Analyzing tinnitus burden (THI) in individuals who received CI in the ipsilateral ear.	Preoperatively, 16 patients had single sided ipsilateral deafness, 2 had bilateral sensorineural hearing loss. 17 of the cases underwent concomitant CI implantation. 13 patients had a reduction in tinnitus burden, 3 cases had no change in and 1 case had worsening of tinnitus. Cochlear implantation had a positive effect on tinnitus burden and hearing outcomes.
Cao et al. [33], 2022	Retrospective Cohort	Otolaryngology	401 patients with VS and tinnitus who underwent RSM or TLM at a single institution.	Understand the changes in tinnitus in VS patients who undergo resection.	Females had more positive tinnitus outcomes than males. The surgical approach (TL v RS) was not significantly associated with tinnitus outcomes. Tumor size of >15 mm was correlated with better tinnitus outcomes. Because the surgical approach was not correlated with the tinnitus outcomes, the tinnitus in VS patients is thought to be of brainstem or CNS control as opposed to a peripheral etiology.
Lin et al. [19], 2023	Retrospective Study	Neurosurgery	32 patients with VS.	VS patients were compared to controls regarding MR images and tinnitus	There was no factor, that was found to be statistically significant regarding changes in tinnitus postoperatively.
Zipfel et al. [34], 2022	Retrospective Study	Neurosurgery	28 patients with VS at less than 21 years of age.	Outcomes in pediatric VS patients following retrosigmoid resection surgery	39.3% of study participants originally had tinnitus. This number dropped to 10.7% postoperatively.
Turek et al. [35], 2023	Prospective Cohort	Neurosurgery	94 patients with intracanalicular VS.	Outcomes in patients after undergoing gamma knife radiosurgery	The number of patients experiencing tinnitus was reduced by the end of the study, however this outcome was not statistically significant.
Bin-Alamer et al. [6], 2023	Retrospective Study	Neurosurgery	267 patients with neurofibromatosis type 2 and VS.	Outcomes in patients following stereotactic radiosurgery	5% patients with tinnitus showed improvement in tinnitus following stereotactic radiosurgery.

## Data Availability

Not applicable.

## Abbreviations

AAO-HNS	American Academy of Otolaryngology–Head and Neck Surgery
cc	cubic centimeter
CI	cochlear implant
CN	cranial nerve
CNS	central nervous system
CPA	cerebellopontine angle
GKS	gamma knife surgery
HA	hearing aid
IAC	internal auditory canal
MF	middle fossa
MRI	magnetic resonance imaging
MS	microsurgery
NF2	Neurofibromatosis Type 2
PRISMA	preferred reporting items for systematic reviews and meta-analyses
ROBINS-I	risk of bias in non-randomized studies—of interventions
RS	retrosigmoid
THI	tinnitus handicap index
TL	translabyrinthine
TLM	translabyrinthine microsurgery
VAS	visual analog scale
VS	vestibular schwannoma
